# Supplementary material for: Discovery of a MUC3B gene reconstructs the membrane mucin gene cluster on human chromosome 7
Source: PLoS One. 2022 Oct 18;17(10):e0275671. doi: 10.1371/journal.pone.0275671 (PMC9578598; doi:10.1371/journal.pone.0275671)
Supplement: S1 File — (DOCX) [file pone.0275671.s007.docx]

**Discovery of a *MUC3B* gene reconstructs the membrane mucin gene cluster on human chromosome 7**

Short title: A *MUC3B* gene on human chromosome 7

Tiange Lang^1^, Thaher Pelaseyed^2^*

1. Big Data Decision Institution, Jinan University, Tianhe, Guangzhou, China.

2. Department of Medical Biochemistry and Cell Biology, Institute of Biomedicine, University of Gothenburg, Gothenburg, Sweden.

* Corresponding author

Email: thaher.pelaseyed@medkem.gu.se (TP)

**
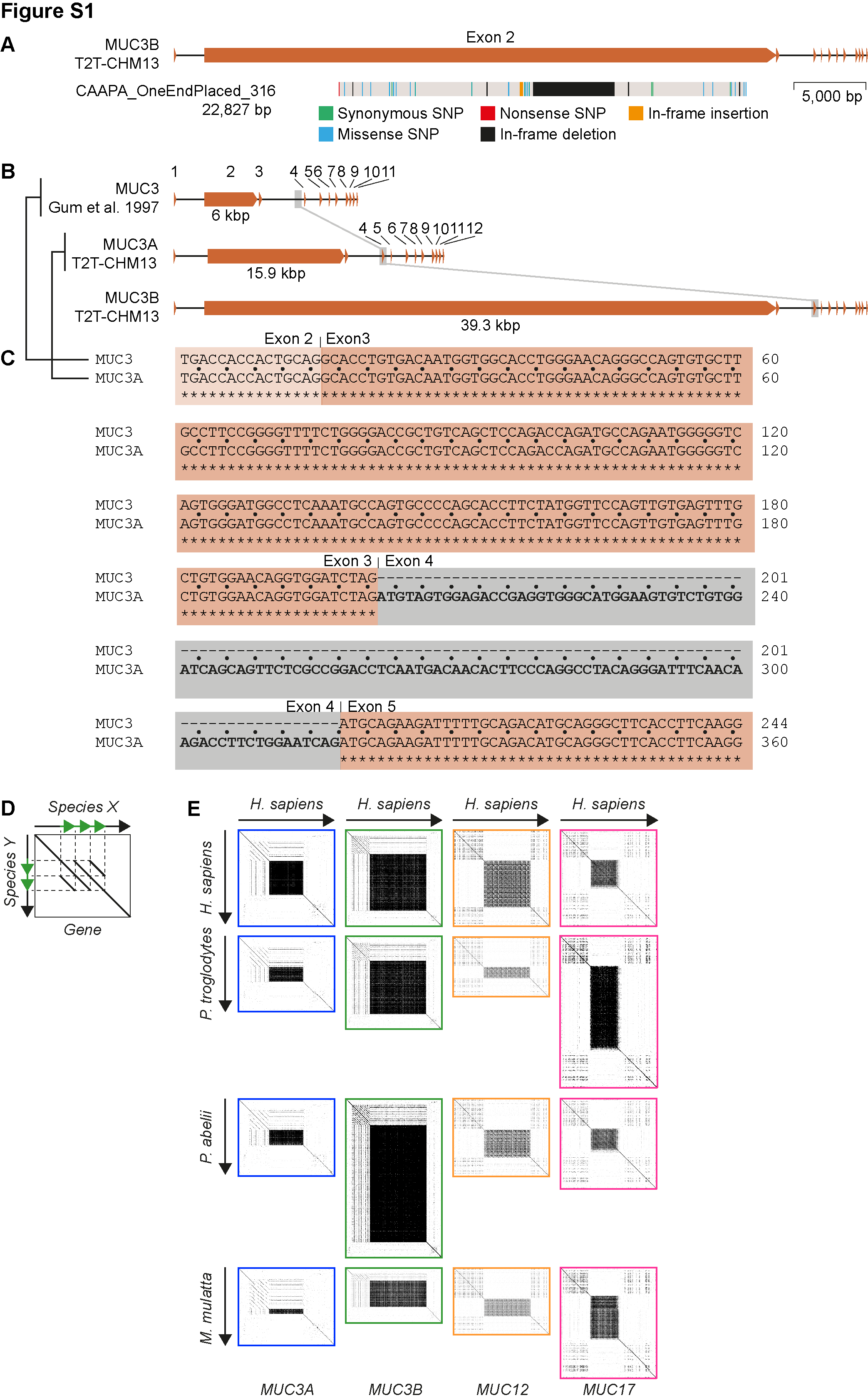
**

**S1 Fig. Exon-intron architecture of *MUC3* genes and dot plots representing pairwise sequence alignments of MUC3 cluster genes belonging to members of the Catarrhini parvorder.** (A) Alignment of contig CAAPA_OneEndPlaced_316 from the African pan-genome [1] to *MUC3B* identified in T2T-CHM13. Synonymous, missense, and nonsense SNPs as well as in-frame deletions and insertions are highlighted. (B) Comparison of the reported exon-intron architecture of *MUC3* [2] with *MUC3A* and *MUC3B* in T2T-CHM13 reveals a new exon 4. (C) Exon sequence alignment showing the previously unresolved exon 4 (gray) in *MUC3A* and *MUC3B*. Black dots indicate the open reading frame. (D) Schematic dot plot depicting pairwise sequence alignment of a gene from two distinct species. Tandem repeat structures are characterized by parallel lines. Differences in tandem repeat number result in asymmetric clusters of parallel lines. (E) Dot plot showing the pairwise alignment of intronic and exonic sequences in MUC3 cluster genes belonging to *H. sapiens* and three Homininae, Ponginae, and Cercopithecinae subfamily species.

**
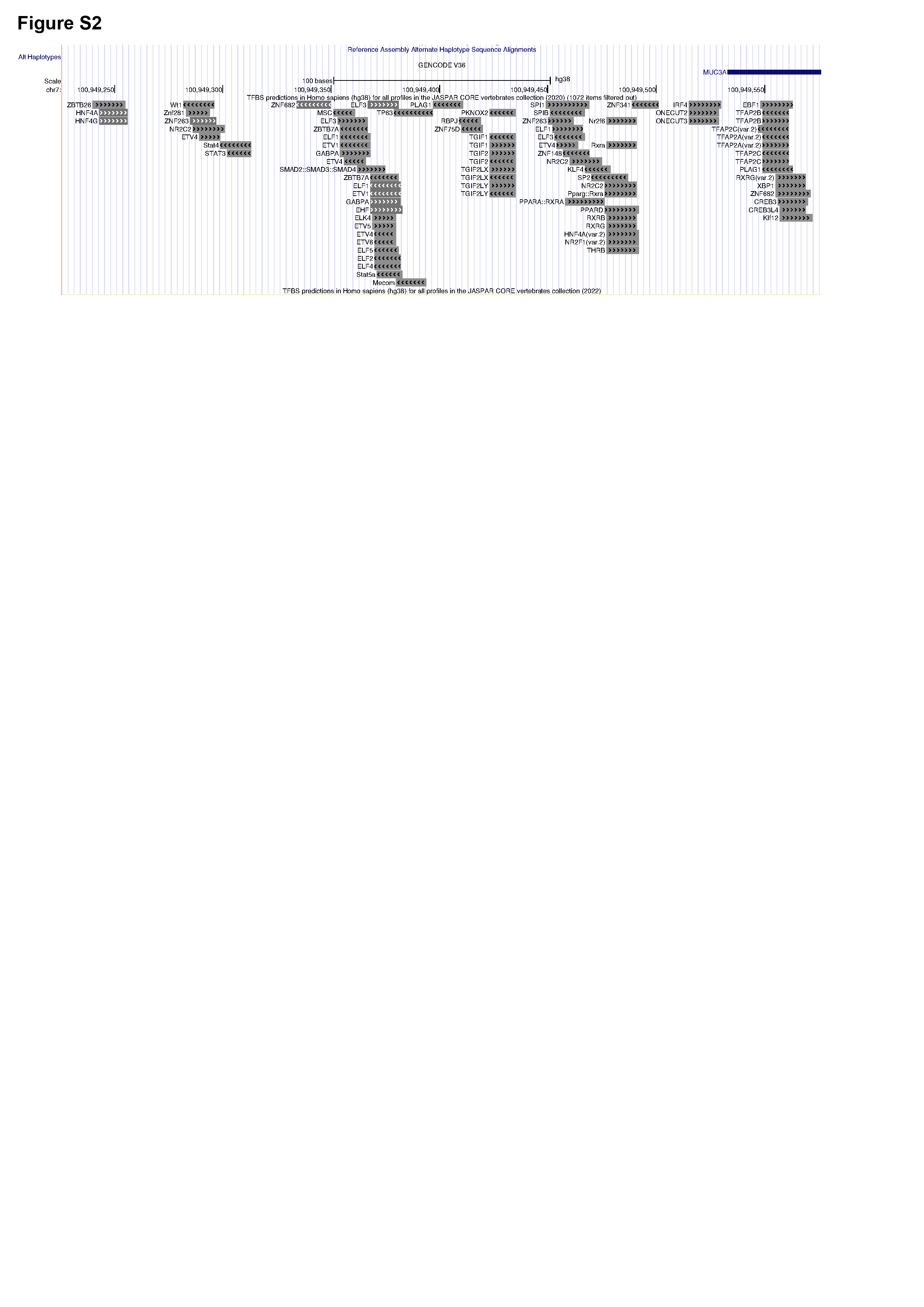
 S2 Fig. Position of transcription factor binding sites upstream of *MUC3A* transcription start site.**

**
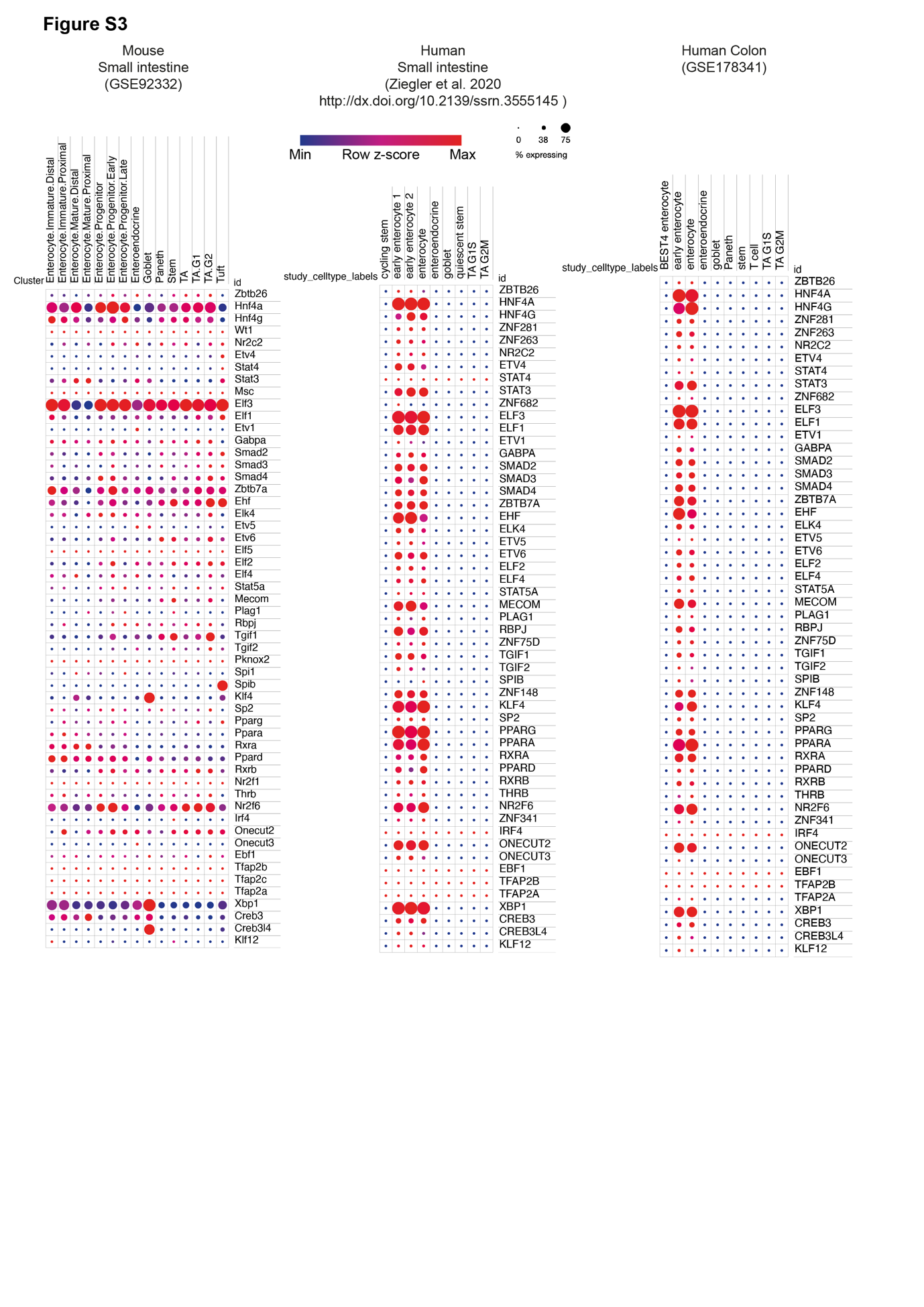
 S3 Fig. Transcription factor expression in human and mouse intestines.** Summary of single-cell gene expression of transcription factors with putative bindings sites upstream of *MUC3A* and *MUC3B* in human small intestine and colon, and mouse small intestine.

**
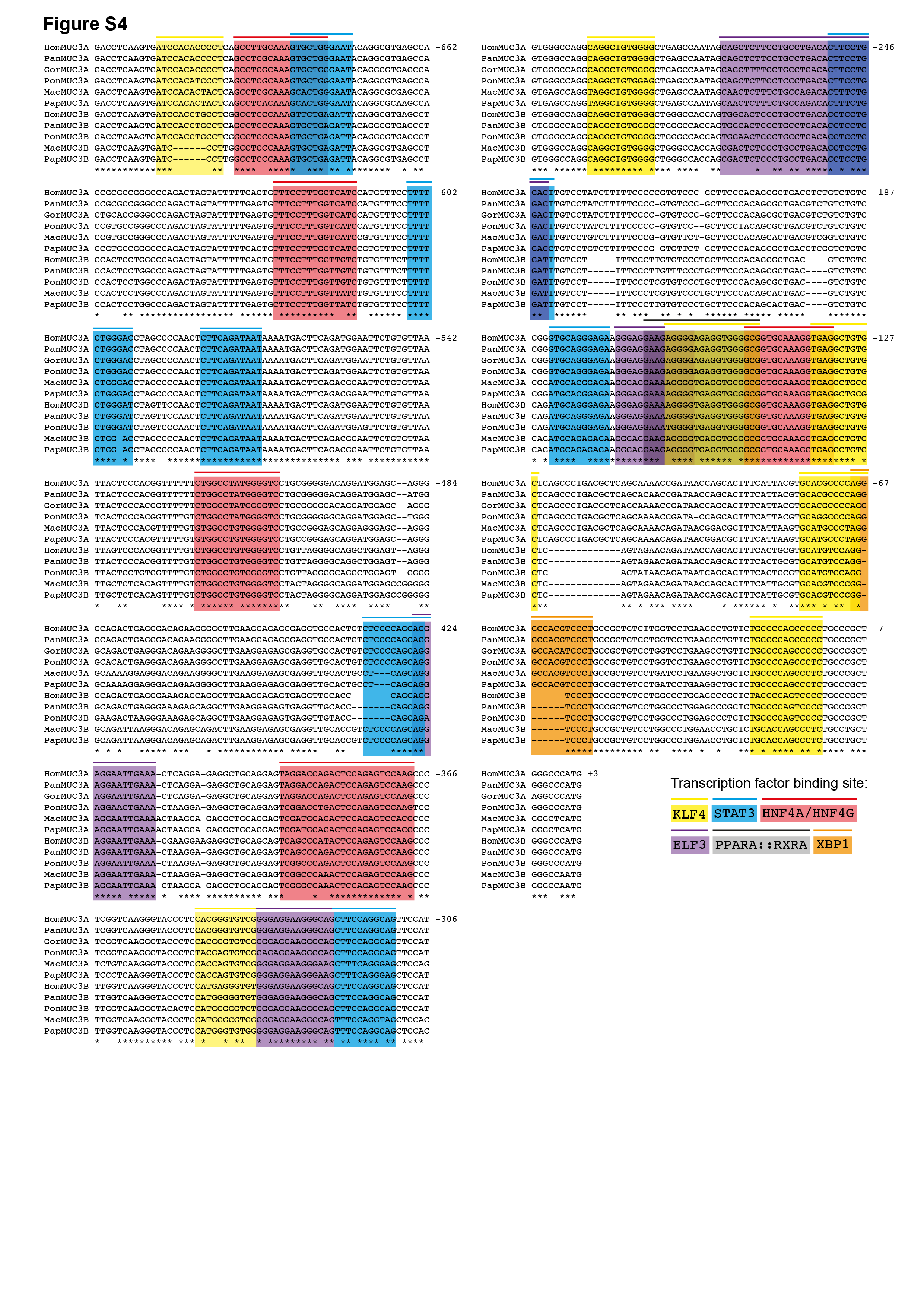
 S4 Fig. Conserved transcription factor binding sites upstream of *MUC3A* and *MUC3B* genes.** Alignment of sequences upstream of *MUC3A* and *MUC3B* shows conservation of binding sites for seven transcription factors expressed in transporting intestinal epithelial cells (IECs) of the human small intestine and colon.

**
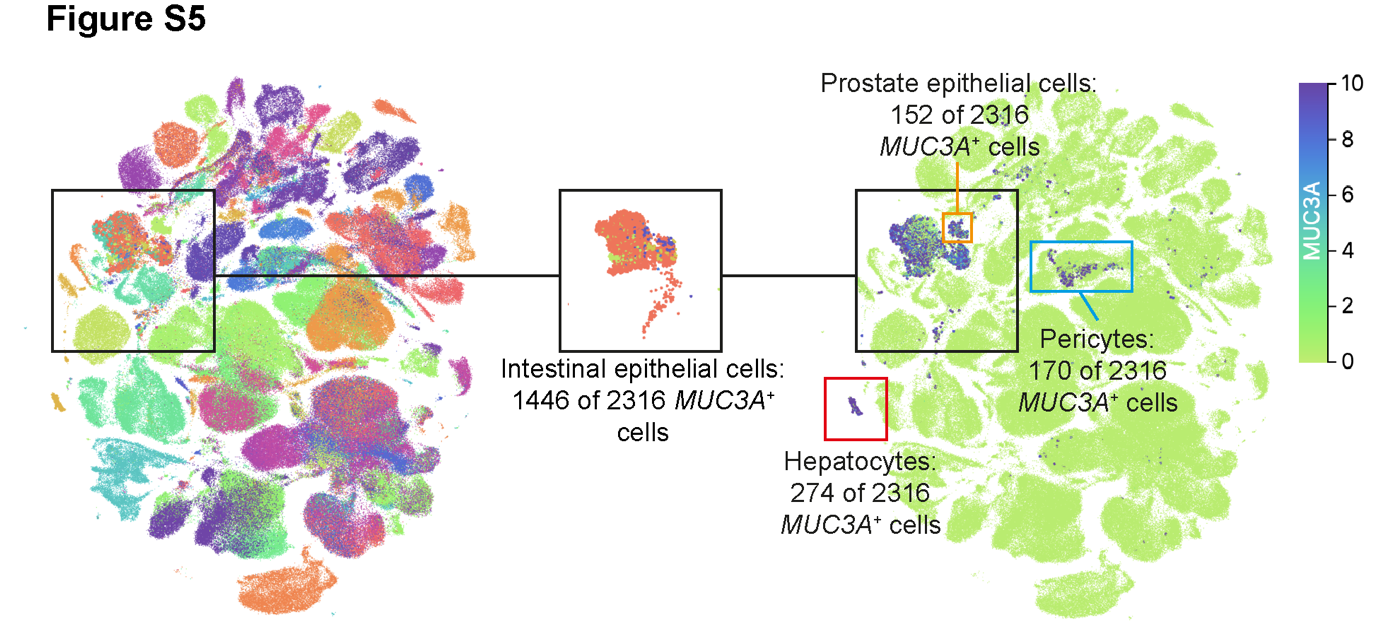
**

**S5 Fig. Expression of *MUC3A* in human cell atlas.** UMAP of 500,000 human cells from 24 organs and 15 individuals, colored according to cell type [3]. IECs constitute the main cell population that expresses *MUC3A*. Scale bar represents normalized gene expression.

**Supplementary methods**

**Perl scripts for extracting specific sequences based on BLAST results**

Perl Script for extraction of fragments using start and end positions obtained by BLAST results

#!/usr/bin/env perl

my $begin = $ARGV[0];

my $end = $ARGV[1];

my $source = $ARGV[2];

open (IN, "<$source") || die "cannnot open \"$source\": $!";

$OneLineSeq = ();

while (<IN>){

chomp;

if (/^>/){print "$_"; print "($begin-$end)\n";}

else {

$OneLineSeq = $OneLineSeq.$_;

$OneLineSeq =~ s/\s//g;

}

}

$result = substr ($OneLineSeq, $begin-1, $end-$begin+1);

print "$result\n";

close IN;

Perl script for extraction of multiple sequences using batch processing based on position file and sequence file

#!/usr/bin/env perl

$posfile = shift;

$seqfile = shift;

$OneLineSeq = ();

open SEQFILE, $seqfile or die "Cannot open file\n";

while (<SEQFILE>){

chomp;

unless (/>/){

$OneLineSeq = $OneLineSeq.$_;

$OneLineSeq =~ s/\s//g;

}

else{$id=$_; $id=~s/ .*//;}

}

close SEQFILE;

open POSFILE, $posfile or die "Cannot open file\n";

while (<POSFILE>){

chomp;

next if /^\s*$/;

@position = split (/\.\./,$_);

$seq = cut ($position[0], $position[1], $OneLineSeq);

print $id,"(",$position[0],"-",$position[1],")\n",$seq,"\n";

}

close POSFILE;

sub cut {

my ($begin, $end, $seq)= @_;

my $result;

$result = substr ($seq, $begin-1, $end-$begin+1);

return $result;

}

Perl scripts used to perform quality control and measure read number in BWA result files

For single end:

#!/usr/bin/env perl

while (<>){

chomp;

next if /^\@/;

@line = split (/\t/,$_);

next if $line[1] >= 256;

print $_,"\n" if $line[4] >= 20;

}

# Read number could be counted by the number of lines of perl result file.

For paired end:

#!/usr/bin/env perl

$k=0;

while (<>){

chomp;

next if /^\@/;

@line = split (/\t/,$_);

$k=$k+1;

if ($k/2!=int($k/2)){$i=1};

if ($k/2==int($k/2)){$i=2};

next if $line[1] >= 256;

$line=$_;

$line=~s/\S+\t//;

print $line[0],"_",$i,"\t",$line,"\n" if $line[4] >= 20;

}

# Read number could be counted by the number of lines of perl result file.

**References**

1. Sherman RM, Forman J, Antonescu V, Puiu D, Daya M, Rafaels N, et al. Assembly of a pan-genome from deep sequencing of 910 humans of African descent. Nat Genet 2018 511. 2018;51: 30–35. doi:10.1038/s41588-018-0273-y

2. Gum JR, Ho JJ, Pratt WS, Hicks JW, Hill AS, Vinall LE, et al. MUC3 human intestinal mucin. Analysis of gene structure, the carboxyl terminus, and a novel upstream repetitive region. J Biol Chem. 1997;272: 26678–86. doi:10.1074/jbc.272.42.26678

3. Jones RC, Karkanias J, Krasnow MA, Pisco AO, Quake SR, Salzman J, et al. The Tabula Sapiens: A multiple-organ, single-cell transcriptomic atlas of humans. Science (80- ). 2022;376. doi:10.1126/science.abl4896
