# Supplementary material for: Discovery of a MUC3B gene reconstructs the membrane mucin gene cluster on human chromosome 7
Source: PLoS One. 2022 Oct 18;17(10):e0275671. doi: 10.1371/journal.pone.0275671 (PMC9578598; doi:10.1371/journal.pone.0275671)
Supplement: S2 File — (PDF) [file pone.0275671.s008.pdf]

S1_raw_images

A. Original uncropped image of Fig 5C, upper panel

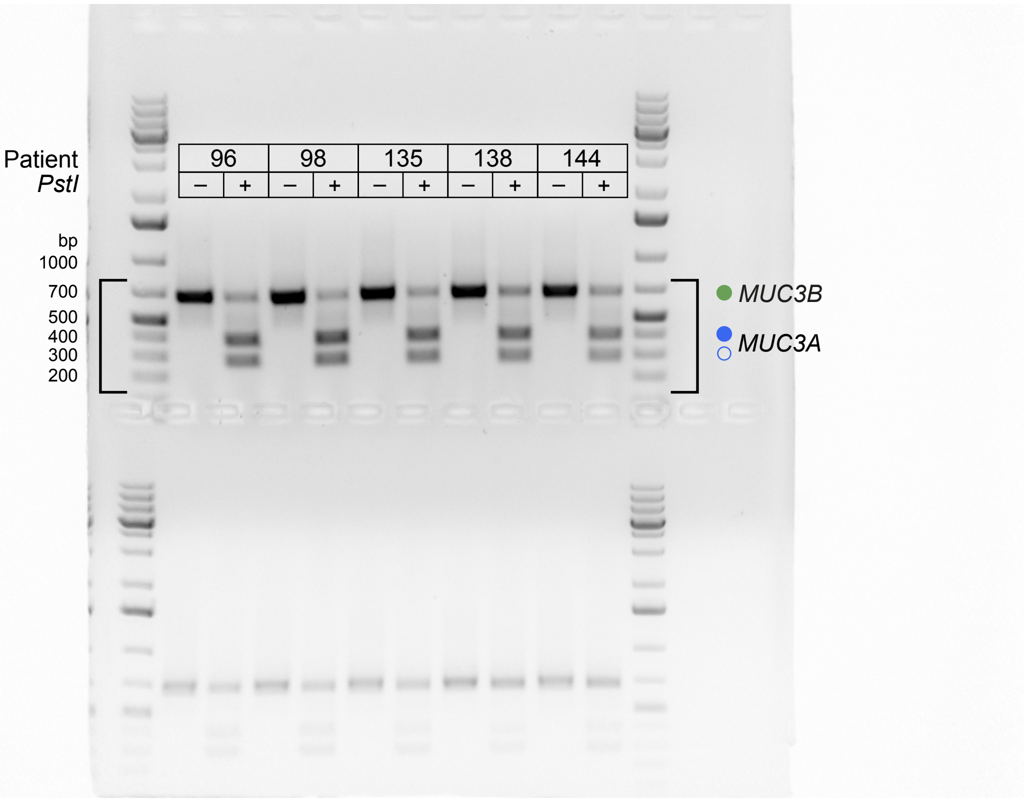

File name: MUCIN85 2021-10-21 13hr 23min.tif
1.5% agarose gel imaged using Bio-Rad Gel Doc EZ Imager

B. Original uncropped image of Fig 5C, lower panel

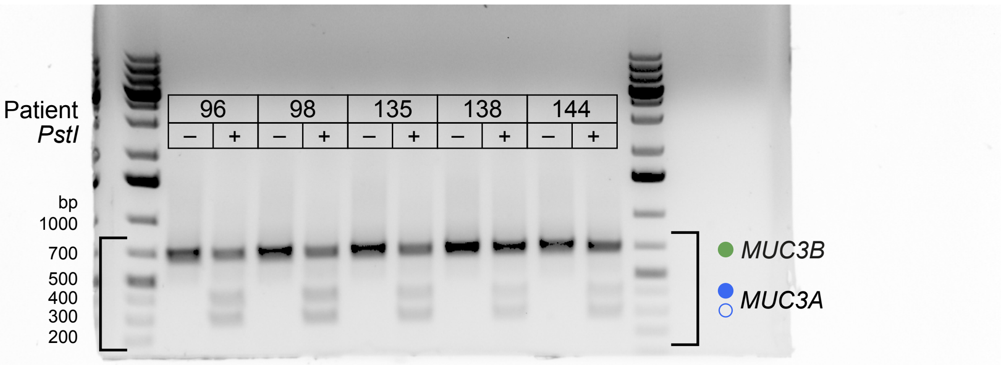

File name: MUCIN85 2021-10-21 13hr 25min.tif
1.5% agarose gel imaged using Bio-Rad Gel Doc EZ Imager
