# Supplementary material for: Discovery of a MUC3B gene reconstructs the membrane mucin gene cluster on human chromosome 7
Source: PLoS One. 2022 Oct 18;17(10):e0275671. doi: 10.1371/journal.pone.0275671 (PMC9578598; doi:10.1371/journal.pone.0275671)
Supplement: Attachment — Submitted filename: Response to reviewers.docx [file pone.0275671.s009.docx]

**Response Reviewers** (see “Revised Manuscript with Track Changes”)

**Reviewer #1**

**Major Comments:**

1. Although the authors are first to better resolve this gene locus using the new T2T human reference genome in an independent study, the authors only briefly stated that MUC3B has been previously found and distinguished from MUC3A:

https://www.sciencedirect.com/science/arNcle/abs/pii/S0006291X00934065?via%3Dihub

https://www.jbc.org/arNcle/S0021-9258(20)75740-6/fulltext.

Recent papers have highlighted this locus as having MUC3-like absence in the hg38 reference genome: https://www.nature.com/arNcles/s41588-018-0273-y.pdf.

These papers should be discussed further as they described MUC3B at the sequence and transcriptomic levels previously. Overall more emphasis should be placed on prior discovery and distinguish the findings of this particular study from previous work.

We thank reviewer #1 for these important comments. We made substantial additions to the original manuscript to address and discuss earlier works by:

Gum JR et al (1997) MUC3 human intestinal mucin. Analysis of gene structure, the carboxyl terminus, and a novel upstream repetitive region. J Biol Chem 272: 26678–86

Pratt WS et al (2000) Multiple Transcripts of MUC3: Evidence for Two Genes, MUC3A and MUC3B. Biochem Biophys Res Commun 275: 916–923

Gum JR et al (2003) Initiation of transcription of the MUC3A human intestinal mucin from a TATA-less promoter and comparison with the MUC3B amino terminus. J Biol Chem 278: 49600–49609

Sherman RM et al (2018) Assembly of a pan-genome from deep sequencing of 910 humans of African descent. Nat Genet 2018 511 51: 30–35

On lines 355-359, we discuss work by Sherman et al (2018). Our sequence alignment shows that contig 316 aligns with exon 2 of *MUC3B* in T2T-CHM13. Our alignment also reveals that contig 316 contains synonymous, missense, and nonsense SNPs as well as in-frame insertions and deletions. The alignment is presented in S1A Fig.

On lines 363-366, we discuss work by Gum et al (1997). The study identified the *MUC3* gene (later named *MUC3A*), encoded by 11 exons. Pratt et al (2000) also identified 11 exons for *MUC3A*. Our study updates the exon-intron architecture of *MUC3A* and *MUC3B*, both of which consists of 12 exons. Importantly, we identified a new exon 4 that was overlooked in the earlier publications. We have added this information in S1B-C Fig.

On lines 427-429, we acknowledge work by Gum et al (2003), in which the *MUC3A* promotor region was mapped to positions -1 - -242 upstream of the transcription start site. Our study complements these findings by adding epigenetic and evolutionary analysis of the promotor regions of *MUC3A* and *MUC3B* in primates.

2. In Line 188 the authors state that the MUC3B “exclusively” evolved in Catarrhini? Was there any sort of BLAST analysis, or syntenic analysis in other species where MUC3B may be ancestral or have evolved recurrently in other species. If not, the language should be toned-down and stated that other species outside of primates were not examined.

We thank reviewer #1 for the valuable comment. We have used BLAST analysis to search for *MUC3B* in species belonging to New World monkeys (Platyrrhini), as well as Scandentia (treeshrew) and Dermoptera (colugos) orders, which share a common ancestor with primates. While we find partial or complete sequences for *MUC3A*, *MUC12*, and *MUC17* in these branches of life, we have not been able to identify any unique sequences belonging to *MUC3B* outside the Catarrhini parvorder. The available genomic sequences for the species outside Catarrhini lack long reads that cover the MUC3 cluster, hampering our efforts to distinguish between *MUC3A* and *MUC3B*. We conclude that available genomes outsides Catarrhini suffer from gaps, as has been observed for human genome assemblies prior to T2T-CHM13. Consequently, we have not been able to determine when during vertebrate mammalian evolution a gene duplication event gave rise to two distinct *MUC3* genes.

We have toned down the language on lines 30-32. We have added the above conclusions on lines 288-294 and lines 590-594.

**Minor Comments:**

1. The author should assess the language more carefully in the manuscript. There are several typos and grammatical issues, particularly in the Discussion section. The examples include but are not limited to the following:

Line 38 : “characterized” is used twice

Line 49: “investigation of” should read “to investigate”

Line 63: “reach” should read “reaches”

Line 229: “in” should be “on”

Line 271: should the second “MUC3A” in the sentence read “MUC3B”?

Line 308: The opening sentence of the Discussion is not a complete sentence.

Line 309: “As results” should read “as a result”

Line 310: “uncomplete” should read “incomplete”

We have corrected all the above mistakes kindly pointed out by the reviewer. In addition, we have carefully assessed the language and corrected the grammar and typos.

2. In the introduction lines 69-81 the authors describe the MUC3 sequence of the mouse as having homology to MUC17. There is no paper cited for this claim.

We apologize for overlooking the reference for this statement. We have added the correct reference in line 100.

**Reviewer #2**

The manuscript entitled “Discovery of a MUC3B gene reconstructs the membrane mucin gene cluster on human chromosome 7” used patients’ tissue from colonoscopy to construct sequence alignment and sequence based on VNTRs in human genome assemblies. The study design was well with valid verification. However, is the novel gene reconstructs specific for intestinal tissues? Or general expression? Please discuss this part in the discussion part.

We thank reviewer #2 for the valuable comment. In the original version of our manuscript, we analyzed single-cell RNA-seq from the human ileum, colon, and rectum as well as kidneys and liver. We found a high number of unique reads for *MUC3A*, *MUC3B*, *MUC12*, and *MUC17* in the ileum, colon, and rectum. None of the genes were expressed in the kidneys and liver (see S3 Table, Tab A). We further experimentally validated mucin gene expression in the human ileum. Our findings are supported by the human cell atlas Tabula sapiens. Single-cell analysis of 500,000 cells from 24 organs of 15 normal human subjects showed that >60% of *MUC3A^+^* cells constitute various populations of intestinal epithelial cells, dominated by transporting epithelial cells (enterocytes). We have added this analysis as a new S5 Fig, and we discuss the findings on lines 478-480.

Notably, our findings contradict previous observations by Kyo K, et al ((2001) J Hum Genet 2001 461 46: 5–20), who detected *MUC3A* transcripts in a broad set of human tissues (heart, liver, prostate, and thymus) using Northern blot. Although we cannot fully explain this discrepancy, our primer-BLAST analysis shows that probes against *MUC3B* were in fact specific for the repetitive exon of *MUC17*. Probes against *MUC3A* were specific but hybridized with exon 2 of *MUC3A*, which is a highly repetitive region with tandem repeats occurring in all members of the mucin gene family. Thus, we can exclude unspecific hybridization to mucin tandem repeat transcripts in the heart, liver, prostate, and thymus. We have inserted our conclusions on lines 480-485 and in S3 Table (tab E).
