# Supplementary material for: Discovery of a MUC3B gene reconstructs the membrane mucin gene cluster on human chromosome 7
Source: PLoS One. 2022 Oct 18;17(10):e0275671. doi: 10.1371/journal.pone.0275671 (PMC9578598; doi:10.1371/journal.pone.0275671)
Supplement: Attachment — Submitted filename: Response to reviewers.docx [file pone.0275671.s010.docx]

**Response Reviewers** (see “Revised Manuscript with Track Changes”)

**Reviewer #1**

The authors have answered my comments to my satisfaction. They have also thoroughly revised the language and grammar in the manuscript, and it reads much better. I have one additional suggestion that I recommend. During the time of this review, two highly relevant publications on mucins were published in high profile journals. It would be wonderful if the authors could cite these two papers in the discussion or introduction. The first proposes a new way mucins evolve (also shows MUC3 locus across species): https://www.science.org/doi/10.1126/sciadv.abm8757 . The other redefines the number of mucins in humans: https://www.nature.com/articles/s41467-022-31062-4.

We thank reviewer #1 for these important comments. We have carefully read the recommended papers.

Pajic et al. (Science Advances 2022) propose convergent evolution as a mechanism by which precursor genes coding for proline-rich proteins gain serine and threonine residues that form mucin-type O-glycosylated exonic repeats. The authors suggest that the retention of novel mucins genes is beneficial for the host as mucins are mechanistic lubricants, serve as matrices enclosing immunological factors, and mediate host-microbe interactions. These arguments are also presented by us in the “Discussion” section. Thus, we have added a reference to Pajic et al. at lines 433-437.

Malaker et al. (Nature Communications 2022) used *in silico* and mucin-selective affinity purification strategies to identify proteins with a putative mucin domain. Expectedly, the reported “mucinome” includes known members of the canonical mucin family (MUC1, MUC4, MUC5AC, MUC5B, MUC6, MUC13, MUC16, and MUC20), whereas the study did not experimentally detect other canonical mucins (MUC2, MUC3A, MUC3B, MUC12, and MUC17) since the analyzed biological samples did not have an intestinal origin. The canonical mucin family is defined by the presence of evolutionarily conserved protein domains that together with an extended and repetitive proline-threonine-serine (PTS)-rich domain construct gel-forming and membrane mucins. The conserved domains include vWD, CysD, and CK domains in gel-forming mucins [1], and SEA or NIDO-AMOP-VWD domains in membrane mucins [2–5].

Notably, many of the detected proteins by Malaker et al. do not carry these conserved domains and exhibit lower PTS content compared to canonical mucins. See table below for the PTS content of a selection of identified proteins.

| **Uniprot** | **Protein** | **PTS (% of total residues)** | **Canonical mucin (YES/NO)** |
| --- | --- | --- | --- |
| P15941 | MUC1 | 44.8 | Yes |
| P98088 | MUC5AC | 45.6 | Yes |
| Q9H3R2 | MUC13 | 30.6 | Yes |
| Q685J3 | MUC17 | 56.5 | Yes |
| O00468 | Agrin | 21.9 | No |
| Q14118 | DAG1 | 20.0 | No |
| Q6WRI0 | IGSF10 | 26.4 | No |
| Q14114 | LRP8 | 19.2 | No |
| Q6ZSS7 | MFSD6 | 21.8 | No |
| P14543 | NID1 | 22.3 | No |
| Q8N131 | Porimin | 35.6 | No |

While the idea of a “mucinome” is conceptually intriguing, we argue for a more stringent definition of what constitutes a mucin [6]. Thus, we have referenced Malaker et al. since the study does not identify novel genes that fulfil the criteria for canonical mucins.

**References**

1.	Trillo-Muyo S, Nilsson HE, Recktenwald C V., Ermund A, Ridley C, Meiss LN, et al. Granule-stored MUC5B mucins are packed by the noncovalent formation of N-terminal head-to-head tetramers. J Biol Chem. 2018;293: 5746. doi:10.1074/jbc.RA117.001014

2.	Moniaux N, Nollet S, Porchet N, Degand P, Laine A, Aubert JP. Complete sequence of the human mucin MUC4: a putative cell membrane-associated mucin. Biochem J. 1999;338: 325. doi:10.1042/0264-6021:3380325

3.	Moniaux N, Escande F, Porchet N, Aubert JP, Batra SK. Structural organization and classification of the human mucin genes. Front Biosci. 2001/10/02. 2001;6: D1192-206. Available: http://www.ncbi.nlm.nih.gov/pubmed/11578969

4.	Ligtenberg MJL, Kruijshaar L, Buijs F, Van Meijer M, Litvinov S V., Hilkens J. Cell-associated episialin is a complex containing two proteins derived from a common precursor. J Biol Chem. 1992;267: 6171–6177. doi:10.1016/S0021-9258(18)42677-4

5.	Macao B, Johansson DG, Hansson GC, Hard T. Autoproteolysis coupled to protein folding in the SEA domain of the membrane-bound MUC1 mucin. Nat Struct Mol Biol. 2006;13: 71–76. Available: http://www.ncbi.nlm.nih.gov/entrez/query.fcgi?cmd=Retrieve&db=PubMed&dopt=Citation&list_uids=16369486

6.	Arike L, Hansson GC. The Densely O-glycosylated MUC2 Mucin Protects the Intestine and Provides Food for the Commensal Bacteria. J Mol Biol. 2016;428: 3221. doi:10.1016/J.JMB.2016.02.010
